# Anxiety Levels in Children with Autism Spectrum Disorder: A Meta-Analysis

**DOI:** 10.1007/s10826-017-0687-7

**Published:** 2017-03-20

**Authors:** Francisca J. A. van Steensel, Emma J. Heeman

**Affiliations:** 1 Child Development and Education, University of Amsterdam, Research priority area Yield, Nieuwe Achtergracht 127, 1018 WS Amsterdam, Netherlands; 2 De Opvoedpoli B.V., Dorpstraat 145, 2712 AG Zoetermeer, Netherlands

**Keywords:** Autism, Anxiety, Children, Meta-analysis

## Abstract

The aim of the current study was to meta-analytically examine whether anxiety levels in children with autism spectrum disorders (ASD) are elevated. A total of 83 articles were selected from a systematic literature search and were included in the meta-analyses. Results demonstrated that children with ASD had higher anxiety levels compared to typically developing children, and this difference increased with IQ. Youth with ASD also tended to have higher anxiety levels compared to clinically referred children, and this difference increased with age. Children with ASD had higher anxiety levels compared to youth with externalizing or developmental problems, but not when compared to youth with internalizing problems. The study findings highlight the importance of more research in order to fully understand the nature and development of anxiety in children with ASD. More specifically, the results suggest that especially high-functioning adolescents with ASD may be at risk for developing anxiety disorders. Therefore, it seems important to carefully follow and monitor children with ASD transcending to adolescence.

## Introduction

Youth with autism spectrum disorders (ASD) are characterized by deficits in social communication and by the presence of restricted interests and repetitive behaviors (APA 2013) but also frequently endorse comorbid symptoms of anxiety, depression, attention and behavior problems (e.g., Gadow et al. 2005; Goldin et al. 2014). Indeed, comorbid disorders are commonly observed in youth with ASD (e.g., de Bruin et al. 2007; Leyfer et al. 2006; Simonoff et al. 2008). Anxiety disorders seem to be one of the most common conditions and are meta-analytically estimated to be prevalent in about 40% of youth with ASD (van Steensel et al. 2011). This prevalence rate is thought to be higher compared to typically developing children in which prevalence rates up to 27% are reported (Costello et al. 2005). In addition, studies comparing anxiety levels of children with ASD to those of typically developing children consistently demonstrate higher anxiety levels for children with ASD (e.g., Kuusikko et al. 2008; Park et al. 2014). However, a systematic (meta-analytical) approach of the size of this difference is currently lacking and therefore we do not know exactly how large this difference is. Similar, there are suggestions that youth with ASD are more prone to anxiety than youth from (some) other clinical groups (e.g., see review of MacNeil et al. 2009), but, again, the size of the possible difference is unknown.

Next to the lack of knowledge about the size of the difference in anxiety between ASD samples and typically developing or clinical samples, it is important to examine which variables may be associated with this difference. For example, the size of the difference may depend on the type of comparison group. Joshi et al. (2010) compared children with ASD to a mixed clinical sample and found that children with ASD were more likely to endorse anxiety disorders (among other disorders). In addition, youth with ASD were found to be more prone for anxiety disorders compared to youth with externalizing disorders; i.e., ADHD (Park et al. 2013; Van Steensel et al. 2013) or conduct disorder (Green et al. 2000; Pugliese et al. 2013). However, when compared to anxiety-disordered children, results are less clear. Farrugia and Hudson (2006) did not report differences between children with ASD and clinically anxious children (child and parent report were aggregated), while Russell and Sofronoff (2005) did find children with ASD to have higher levels of anxiety according to parent report. Further, van Steensel et al. (2012) found that parents reported higher anxiety symptoms (total, specific, social, generalized and panic anxiety) for children with ASD compared to clinically anxious children, however, child report did not reveal any group differences except for specific phobias. Thus, next to the type of comparison group, type of informant (e.g., parent, child, teacher, clinician report) may be an important factor to consider. Other factors of interest are age and IQ. It is known that anxiety tends to increase with age in general, and it seems that the prevalence of anxiety disorders in youth with ASD follows a similar developmental course (van Steensel et al. 2011). However, what is not known is whether the difference in anxiety levels between children with ASD and typically developing children, or between children with ASD and clinically referred children, is the same or different across developmental phases. The same applies for IQ, which may be even more complicated because the relation between IQ and anxiety in youth with ASD is not clear itself. That is, some studies found a positive association between IQ and anxiety (e.g., Gadow et al. 2005; Eussen et al. 2013), while others point to a negative association (e.g., White and Roberson-Nay 2009) or suggest a quadratic instead of a linear relation (van Steensel et al. 2011).

The aim of the current study was to meta-analytically estimate the difference in anxiety levels between children with ASD and typically developing children, and between children with ASD and clinically referred children. As previous studies point to different findings, and results seem to be depending on the type of comparison group and informant, additional analyses were conducted for the different comparisons groups (internalizing, externalizing, and other problems) and informants (parents and children). Further, the moderating influence of age and IQ was examined by including these variables as moderators.

## Method

### Literature Search

The literature search was conducted in two phases. The first phase covered articles published till the half of 2010 leading to a selection of 86 studies that reported about an ASD sample and an anxiety measure (procedure and results of this first phase are reported in van Steensel et al. 2011). The second phase of the literature search covered the period 2010 till 2016. The same approach for the literature search was used as in the first phase: the words “Autism” “High Functioning Autism” “Asperger” “Pervasive Developmental Disorder” “PDD” were used in combination with “Anxiety” “Anxious” “Anxiety Disorder” “Comorbidity” “Comorbid disorder” “Psychological disorder” and “Psychiatric disorder”, within the following databases: ‘PsychInfo’, ‘Pubmed’, ‘Web of Science’ and ‘ERIC’. During both phases, abstracts were screened for relevance and got selected if they reported about (1) an ASD sample and (2) a measure assessing anxiety problems (which did not explicitly needed to be mentioned in the articles; e.g., measures assessing comorbidity were also selected at this point). During the second literature search, 7332 abstracts were screened of which over 10% was screened by both authors (agreement between the authors regarding the selection of articles was over 98% and in case of disagreement articles got fully screened). Full articles (*N* = 184) were then screened and included in the meta-analysis if (1) an ASD sample and a comparison group was included, (2) anxiety was measured by a standardized questionnaire, (3) a mean age <19 years was reported, and (4) articles were published in English. Studies were excluded if (1) they did not report on empirical data (i.e., articles were reviews), (2) they reported about case studies only, (3) other informant reports than child or parent (e.g., only teacher report) was used, or (4) they used an interview (measuring anxiety disorders) instead of a questionnaire (measuring anxiety levels). The later exclusion criteria was based on the dimensional focus of the current meta-analyses (instead of the focus on a categorical approach as was done in van Steensel et al. 2011). A total of 83 studies were found to meet the criteria and were selected for the current meta-analyses (see Table 1).

**Table 1:** Overview of the studies included in the meta-analyses

Author (year)	Anxiety measure	ASD measure	ASD group			Comparison group	*d*_ASD vs. comparison_		
			*N*	*M*_*age*_	*M*_*IQ*_		Parent	Child	*M*_*parent+child*_
Bauminger et al. (2010)	Child Behavior Checklist (CBCL)	ADI-R	43	10.06	105.65	Typically	0.98	–	0.98
Bellini (2004)	*Parent report:* Behavioral Assessment System for Children (BASC) *Child report:* Multidimensional Anxiety Scale for Children (MASC)	Clinicians	41	14.22	99.94	Normative	1.82	0.95	1.39
Blakeley-Smith et al. (2012)	Screen for Child Anxiety Related Emotional Disorders (SCARED)	ADOS and ADI-R or SCQ	63	10.10	107.00	Anxiety/mood	0.81	0.22	0.50
Bitsika and Sharpley (2015)	Child and adolescent symptom inventory (CASI)	Clinicians	140	11.16	96.08	Community	–	0.95	0.95
Boulter et al. (2014)	Spence Children’s Anxiety Scale (SCAS)	ADOS (USA sample), multi-disciplinary team (UK sample)	114	12.70	108.50	Typically	1.60	0.37	0.99
Bradley et al. (2004)	Diagnostic Assessment for the Severely Handicapped-II (DASH-II)	ADI-R	12	16.30	<70	ID	1.06	–	1.06
Brereton et al. (2006)	Developmental Behavioral Checklist (DBC-P)	Clinicians (inter-rater); ABC and CARS	381	7.38	<70	ID	0.51	–	0.51
Burnette et al. (2005)	Behavioral Assessment System for Children (BASC)	ASSQ and ASAS	31	11.25	109.74	Typically/LD	–	0.11	0.11
Chamberlain et al. (2013)	Spence Children’s Anxiety Scale (SCAS)	ADOS and SRS	18	16.64	104.82	Typically	1.17	0.39	0.78
Cholemkery et al. (2014a)	Parent questionnaire from the Diagnostic System for Mental Disorders in	ADI-R and ADOS	55	12.50	100.60	Typically	1.57	–	1.57
	Children and Adolescents (DISYPS-II)					ODD/CD	0.25	–	0.25
Cholemkery	Parent questionnaire from the Diagnostic System for Mental	ADI-R and ADOS	60	12.28	102.15	Typically	1.60	–	1.60
(2014b)	Disorders in Children and Adolescents (DISYPS-II)					Selective mutism	−0.66	–	−0.66
						Social anxious	−0.47	–	−0.47
Clawson et al. (2014)	Screen for Child Anxiety Related Emotional Disorders (SCARED)	ADOS and SCQ	38	13.10	107.70	Typically	1.12	–	1.12
Davis et al. (2010)	Autism Spectrum Disorders—Comorbidity for Children (ASD-CC)	Clinicians (inter-rater)	66	7.46	>70	Typically	1.55	–	1.55
Dewrang and Sandberg (2011)	Beck Anxiety Inventory (BAI)	Clinicians	24	18.54	>70	Typically	–	0.22	0.22
Farrugia and	Spence Children’s Anxiety Scale (SCAS)	Clinicians	29	13.76	>70	Non-clinical	–	–	1.15
Hudson (2006)						Anxious	–	–	0.21
Gadow et al. (2004)	Early Child Inventory-4 (ECI-4)	Clinicians (inter-rater)	172	4.20	79.00	Regular education	0.22	–	0.22
						Special education	0.32	–	0.32
						Clinical	0.18	–	0.18
Gadow et al. (2005)	Child Symptom Inventory (CSI)	Clinicians (inter-rater)	284	8.30	92.00	Regular education	0.73	–	0.73
						Special education	0.51	–	0.51
						Clinical	0.16	–	0.16
Gadow et al.	Child Symptom Inventory (CSI)	Clinicians (inter-rater)	87	8.50	96.90	ADHD	0.40	–	0.40
(2009)						ADHD + tic	−0.30	–	−0.30
Gillott et al.	*Parent report:* Social Worries Questionnaire (SWQ)	Clinicians	15	10.27	>70	Typically	2.06	0.93	1.50
(2001)	*Child report:* Spence Children’s Anxiety Scale (SCAS)					Language impairment	1.36	0.90	1.13
Goldin et al.	Behavioral Assessment System for Children, Second Edition	Clinician	57	7.4	NA	Typically	−0.27	–	−0.27
(2014)	(BASC-2)					Atypically	−0.58	–	−0.58
Green et al. (2013)	Child Behavior CheckList (CBCL)	ADI-R and ADOS	25	13.10	101.16	Typically	1.31	–	1.31
Greenaway and Howlin (2010)	Spence Children’s Anxiety Scale (SCAS)	SCQ	41	13.20	100.00	Typically	1.92	1.07	1.50
Griffith et al. (2010)	Reiss Scales for Children’s Dual Diagnosis	Parent-reported diagnosis	19	10.16	<70	Down syndrome	1.48	–	1.48
						Mixed disorders	0.98	–	0.98
Guttmann-	Child Symptom Inventory (CSI)	Clinicians (inter-rater)	204	8.70	99.41	Community	0.50	–	0.50
Steinmetz et al.						ADHD	0.22	–	0.22
(2010)						ADHD + tic	−0.05	–	−0.05
Guy et al. (2014)	Screen for Child Anxiety Related Emotional Disorders (SCARED)	ADI-R and ADOS	14	12.27	101.14	Typically	1.38	–	1.38
Hallett et al.	Revised Child Anxiety and Depression Scale (RCADS)	CAST and DAWBA	142	13.50	88.07	Control twins	0.94	0.34	0.64
(2013)						Unaffected twin	0.68	0.25	0.47
						Broader autism	0.15	0.13	0.14
Hebron and	Beck Youth Inventories, 2^nd^ Edition (BYI-II)	Confirmed diagnosis but	22	14.17	NA	Typically	–	1.10	1.10
Humphrey (2014)		not reported what measure was used				Dyslexia	–	0.81	0.81
Hollocks et al. (2013)	Spence Children’s Anxiety Scale (SCAS)	SCQ	38	12.90	95.60	Typically	1.64	2.06	1.85
Hollocks et al. (2014)	Spence Children’s Anxiety Scale (SCAS)	ADI-R and/or ADOS and/or SCQ	52	12.84	101.15	Typically	1.98	1.47	1.73
Jarrold et al. (2013)	Multidimensional Anxiety Scale for Children (MASC)	ASSQ, SCQ, SRS	37	11.97	108.10	Typically	–	0.72	0.72
Kim et al. (2000)	Revised Ontario Child Health Study (OCHS) questionnaire	ADI-R	59	12.00	>70	Normative	0.55	–	0.55
Kim et al. (2014)	*Parent report:* Behavior Assessment System for Children (BASC) *Child report:* Manifest Anxiety Scale for Children (MASC)	ADI-R	19	11.50	115.20	Typically	2.19	0.43	1.31
Kline et al. (1987)	Personality Inventory for Children (PIC)	Clinicians	52	9.62	<70	ID	0.22	–	0.22
Kuusikko et al. (2008)	*Parent report:* Child Behavior CheckList (CBCL) *Child report:* Social Anxiety Scale for Children-Revised (SAS-C) and Social Phobia and Anxiety Inventory for Children (SPAI-C)	ADI-R and ADOS	45	11.20	≥ 80	Community	1.45	0.75	1.10
Lawson et al. (2015)	Child Behavior CheckList (CBCL)	ADI-R and/or ADOS	70	10.07	107.01	ADHD	−0.09	–	−0.09
Lopata et al. (2010)	Behavioral Assessment System for Children (BASC)	ADI-R	40	9.75	110.14	Typically	0.81	0.05	0.43
Mahan and Matson (2011)	Behavioral Assessment System for Children (BASC)	DSM-IV/ICD-10 checklist	38	9.53	NA	Typically	−0.16	–	−0.16
Matson and Love (1990)	Revised Fear Survey Schedule for Children (FSSC-R)	No confirmation of diagnosis	14	9.54	NA	Typically	−0.81	–	−0.81
May et al. (2014)	Spence Children’s Anxiety Scale (SCAS)	SRS	56	9.88	96.15	Typically	1.46	–	1.46
May et al. (2015a)	Spence Children’s Anxiety Scale (SCAS)	SRS	45	10.27	99.20	Typically	1.38	–	1.38
						Anxiety	−0.33	–	−0.33
May et al. (2015b)	Spence Children’s Anxiety Scale (SCAS)	SRS	44	10.39	100.70	Typically	1.50	0.12	0.81
May et al. (2015c)	Spence Children’s Anxiety Scale (SCAS)	SRS	46	10.38	95.14	Typically	1.51	–	1.51
Mazefsky et al. (2011)	Child Behavior CheckList (CBCL)	ADI-R and ADOS	108	12.13	100.53	Community	1.87	–	1.87
Mazefsky et al. (2014)	Screen for Child Anxiety Related Emotional Disorders (SCARED)	ADI-R and ADOS	25	15.22	110.48	Typically	1.60	0.42	1.01
Mazzone et al.	*Parent report:* Child Behavior Checklist (CBCL)	ADOS	30	11.06	117.72	Typically	1.48	0.72	1.10
(2013)	*Child report:* Multidimensional Anxiety Scale for Children (MASC)					Depressive	−0.41	0.32	−0.05
Melfsen et al.	Social Phobia and Anxiety Inventory for Children (SPAIK)	Two psychiatrics (both	7	13.71	>70	Normative	–	1.04	1.04
(2006)		had to confirm ASD)				Clinical	–	0.53	0.53
						Social anxious	–	−0.84	−0.84
Meyer et al. (2006)	Behavioral Assessment System for Children (BASC)	ASAS, ASSQ	31	10.10	>70	Typically	0.84	0.36	0.60
Mikita et al. (2015)	Spence Child Anxiety Scale (SCAS)	ADI-R and/or ADOS	52	12.80	101.20	Typically	1.68	1.40	1.54
Miller et al. (2013)	Behavioral Assessment System for Children (BASC)	ADOS and SCQ	40	12.03	102.29	Typically	1.57	–	1.57
Muratori et al. (2011)	Child Behavior CheckList 1.5–5 (CBCL)	ADOS and SCQ	101	3.67	NA	Typically	0.56	–	0.56
						Clinical	−0.43	–	−0.43
Ooi et al. (2011)	Child Behavior Checklist (CBCL)	Clinicians	86	9.80	>70	Anxiety	−0.65	–	−0.65
Ozsivadjian et al. 2012	Spence Children’s Anxiety Scale (SCAS)	SCQ	30	13.00	94.90	Typically	1.57	0.67	1.12
Oswald et al. (2016)	*Parent report:* Revised Child Anxiety and Depression Scale (RCADS) *Child report:* Multidimensional Anxiety Scale for Children (MASC)	AQ	32	14.86	105.38	Typically	1.13	0.48	0.80
Park et al. (2013)	*Parent report:* Child Behavior CheckList (CBCL)	ADI-R and ADOS	56	9.39	114.11	Depressive	−0.28	−0.08	−0.18
	*Child report:* State-Trait Anxiety Inventory for Children (STAIC)					ADHD	0.56	0.22	0.39
Park et al. (2014)	Behavioral Assessment System for Children, Second Edition (BASC-2)	ADOS, ADI-R and ASSQ	87	8.99	90.97	Community	0.72	–	0.72
						Clinical	0.01	–	0.01
Piper et al. (2014)	Brief Problem Monitor, (abbreviated form of the CBCL)	No confirmation of diagnosis	23	11.50	NA	Typically	11.74	–	11.74
						ADHD	1.54	–	1.54
						Depression	−4.06	–	−4.06
						Anxiety	−3.38	–	−3.38
						Bipolar	−2.48	–	−2.48
						Attachment	−0.73	–	−0.73
						Developmental	−0.50	–	−0.50
						Speech delay	1.89	–	1.89
Rescorla et al. (2014)	Child Behavior Checklist 1.5–5 (CBCL)	Clinicians (both had to	46	3.54	NA	Typically	0.29	–	0.29
		confirm diagnosis)				DD	−0.11	–	−0.11
						Clinical	−0.16	–	−0.16
Predescu et al. (2013)	Child Behavior Checklist 1.5–5 (CBCL)	KID-SCID	103	4.53	NA	Typically	1.05	–	1.05
						DD	−0.38	–	−0.38
						ADHD	0.05	–	0.05
Pugliese et al. (2013)	Multidimensional Anxiety Scale for Children (MASC)	Clinicians	30	11.75	93.75	Social anxious	–	−0.30	−0.30
						ODD/CD	–	0.68	0.68
Richdale et al. (2014)	Depression, Anxiety and Stress Scale (DASS-21)	SCQ	27	15.5	>70	Typically	–	0.60	0.60
Richdale and Baglin (2015)	*Parent report:* Child Behavior CheckList (CBCL) *Child report:* Screen for Childhood Anxiety Related Emotional Disorders (SCARED)	SCQ	17	10.03	>70	Typically	2.31	0.51	1.41
Rieske et al. (2012)	Autism Spectrum Disorder—Comorbidity for Children (ASD-CC)	Clinicians	108	8.66	>70	Typically	1.55	–	1.55
Rodgers et al. (2012)	Spence Children’s Anxiety Scale (SCAS)	SCQ	34	12.17	97.02	Williams	0.43	–	0.43
Russell and Sofronoff (2005)	Spence Children’s Anxiety Scale (SCAS)	CAST	65	10–13	>70	Normative	2.44	0.70	1.57
						Anxiety	0.46	0.00	0.23
Samson et al. (2015)	Child Behavior CheckList (CBCL)	ADI-R and ADOS	32	12.66	104.31	Typically	1.20	–	1.20
Schroeder et al. (2011)	Child Behavior CheckList (CBCL)	KADI	15	12.07	115.40	Normative	1.73	–	1.73
Schwenk and Freitag (2014)	Child Behavior CheckList (CBCL)	ADI-R and/or ADOS	60	12.40	102.59	Typically	1.17	–	1.17
						ADHD	0.44	–	0.44
Sharma et al. (2014)	Spence Children’s Anxiety Scale (SCAS)	CAST	22	9.64	>70	Typically	0.99	0.99	0.99
Simon and Corbett (2013)	Multidimensional Anxiety Scale for Children (MASC)	ADOS	19	9.99	98.56	Typically	1.04	–	1.04
Skokauskas and Gallagher (2012)	Child Behavior CheckList (CBCL)	ADI-R and/or ADOS	67	12.73	61.03	Typically /ID	−0.12	–	−0.12
Solomon et al. (2012)	Behavioral Assessment System for Children (BASC)	ADOS and SCQ	40	12.23	104.08	Typically	1.01	–	1.01
Stein et al. (2014)	Child and Adolescent Symptom Inventory (CASI)	ADOS	22	8.20	NA	Typically	1.49	–	1.49
Sterling et al. (2013)	*Parent report:* Child Behavior CheckList (CBCL) *Child report:* Revised Children’s Manifest Anxiety Scale Second Edition (RCMAS-2)	ADI-R and ADOS	20	14.45	104.60	Typically	1.37	−0.39	0.49
Stoppelbein et al. (2016)	Child Behavior CheckList (CBCL)	ADI-R and/or ADOS	45	8.43	NA	Typically	2.00	–	2.00
Swartz et al. (2013)	Spence Children’s Anxiety Scale (SCAS)	ADI-R and ADOS	32	13.57	113.40	Typically	–	0.68	0.68
Schwenk and Freitag (2014)	Child Behavior CheckList (CBCL)	ADI-R and/or ADOS	60	12.40	102.59	Typically	1.17	–	1.17
						ADHD	0.44	–	0.44
Thede and Coolidge (2007)	Coolidge Personality and Neuropsychological Inventory (CPNI)	Clinician and a 44-item survey developed by the authors	31	10.80	>70	Normative	0.75	–	0.75
Thurman et al. (2014)	Anxiety, Depression, and Mood Scale (ADAMS)	ADOS	41	7.17	61.85	Fragile X	−0.52	–	−0.52
Tonge and Einfeld (2003)	Developmental Behavior Checklist	Clinicians (inter-rater)	118	8.48	<70	ID	0.41	–	0.41
van Rijn et al. (2014)	Social Anxiety Scale (SAS)	ADI-R	58	11.97	101.65	Typically	–	0.23	0.23
						Extra X	–	−0.52	−0.52
Van Steensel et al. (2012)	Screen for Child Anxiety Related Emotional Disorders (SCARED)	ADI-R	115	11.37	>70	Anxiety	0.51	0.00	0.26
von Gontard et al. (2015)	Child Behavior CheckList (CBCL)	ADI-R and ADOS	40	11.30	102.20	Typically	1.66	–	1.66
White et al. (2015b)	Social Worries Questionnaire (SWQ)	ADI-R and/or ADOS	15	14.88	>70	Typically	0.66	0.40	0.53
Williamson et al. (2008)	Spence Children’s Anxiety Scale (SCAS)	Clinicians	19	13.17	>70	Typically	–	0.64	0.64

*ABC* Autism Behavior Checklist, *ADI-R* Autism Diagnostic Interview- Revised, *ADOS* Autism Diagnostic Observation Schedule, *ASAS* Australian Scale for Asperger Syndrome, *ASDS* Asperger’s Syndrome Diagnostic Scale, *ASSQ* Autism Spectrum Screening Questionnaire, *AQ* Autism Spectrum Quotient, *CARS* Childhood Autism Rating Scale, *CAST* Childhood Autism Spectrum Test, *CD* Conduct Disorder, *DAWBA* Development and Well-Being Assessment, *DD* Developmental Delay, *ID* Intellectual Disability, *KADI* Krug Asperger’s Disorder Index, *LD* Learning Disability, *ODD* Oppositional Defiant Disorder, *SCQ* Social Communication Questionnaire, *SRS* Social Responsiveness Scale

### Coding

Mean age, mean IQ, informant (child and/or parent) and comparison group was coded for all studies (see Table 1) by the authors of the study, and a graduate student double coded 10%. The interrater agreement was excellent (above .90). For analyses, the comparison group was divided into: (1) typically developing children, and (2) clinically referred children. The clinical comparison group was further subdivided in: (2a) internalizing problems (anxiety and/or mood problems), (2b) externalizing problems (ADHD, oppositional defiant disorder, and/or conduct disorder), (2c) developmental problems (e.g., Down-Syndrome, Fragile X, intellectual/language disabilities, learning problems), or (2d) clinical not otherwise specified (mixed clinical sample or special education sample). Two studies (Burnette et al. 2005; Skokauskas and Gallagher 2012) used a mixed sample consisting of typically developing children as well as children with learning/intellectual disabilities and were therefore registered as having both a typically developing as well as a clinical comparison group.

For all studies an effect size (Cohen’s *d*) of the difference in anxiety levels between the ASD sample and the comparison sample was calculated by the authors of the study. If both parent and child report was used, a separate effect size for parent report and for child report was calculated. However, for the main analyses, a mean of the parent and child effect size was taken to represent an average effect size for that study. The rationale for aggregating child- and parent report was that there are many studies suggesting to use multi-informant reports for measuring childhood anxiety, and that—while child-parent agreement is only modest at best (also in non-ASD samples)—child report, in addition to parent report, may provide important information from another perspective (e.g., Brown-Jacobsen et al. 2011; Comer and Kendall 2004; Grills and Ollendick 2003). Furthermore, we reasoned that the aggregation of child-and parent reports would result in a best-estimate of the ‘real truth’ because whereas children with ASD may underreport their anxiety levels (e.g., due to disabilities in insight), parents may over-report anxiety levels of their children (e.g., due to being unable to disentangle anxiety symptoms from ASD symptoms) (van Steensel et al. 2011). Finally, the aggregation of child- and parent reports allowed for a larger number of studies to be included in the analyses (which was particularly needed to compare the ASD sample to the specific comparisons groups). A similar approach of aggregation was used when multiple comparison groups were used in a single study: i.e., effect sizes between the ASD sample and each of the comparison groups were calculated separately and then a mean of these effect sizes was taken to represent an average effect size of that particular study. In addition, some studies used (part of) the same ASD sample in multiple studies (e.g., Cholemkery et al. 2014a, b). In those cases, a mean effect size of the overlapping studies was calculated and entered in the meta-analysis.

When multiple measures in a study were used to assess anxiety; (1) a total anxiety score was chosen over a specific anxiety score (e.g., social anxiety or generalized anxiety), (2) questionnaires measuring anxiety were preferred over a general problem behavior questionnaire, however, (3) a total anxiety score of a general problem behavior questionnaire was preferred over a specific anxiety score (e.g., a questionnaire measuring social anxiety only). Some studies reported about specific anxiety scores only (i.e., no total anxiety score but only subscale scores for social anxiety, generalized anxiety etc.) and in those cases, a mean effect size was calculated from the specific anxiety scores.

### Outliers, Normality and Publication Bias

For the two main analyses (ASD compared to typically developing and ASD compared to clinical), normality, outliers and publication bias was examined. Using standardized Z-scores, one outlier was identified in the analyses comparing youth with ASD to typically developing children. This outlier was adjusted in the highest score not being an outlier. Normality was explored with the Kolmogorov-Smirnov test, and no violations were found. Publication bias was examined by statistically testing for funnel plot asymmetry with the rank order correlation coefficient, the Egger’s regression method and by adding the standard error as a moderator to the random effect model. All methods revealed non-significant results.

### Analyses

Two main meta-analyses were conducted: (1) ASD vs. typically developing children, and (2) ASD vs. clinically referred children. The aggregated parent + child effect sizes were used for these analyses. Moderator analyses were conducted for both meta-analyses with mean age and mean IQ entered as predictors. In addition, separate meta-analyses were conducted for parent and child report, and for the different comparison groups (internalizing problems, externalizing problems, developmental problems, and clinical not otherwise specified). The SPSS macro’s of Lipsey and Wilson were used for analysis, and homogeneity across studies was examined with Q statistics.

In meta-analyses, both fixed as well as random models can be conducted. Hedges and Vevea (1998) argue that while in some cases the choice for either model is led by the homogeneity (or heterogeneity) of the effect-size parameter (i.e., fixed models are used when the parameter estimates of the selected studies are homogeneous, while random models are used when evidence of heterogeneity is found), the choice of the model should depend on the interferences one wants to make. That is, if one wants to make interferences about the selected studies then a fixed model approach is appropriate, while as one wants to make interferences beyond the selected studies then one should use a random model approach (Hedges and Vevea 1998).

## Results

Table 2 displays the results of the meta-analyses. Homogeneity tests were significant for all meta-analyses indicating substantial heterogeneity across studies, and therefore moderators (mean age and mean IQ) were analyzed using the random effects model.

**Table 2:** Results of the meta-analyses comparing the anxiety levels of youth with ASD to typical developing and clinically referred youth

	*k*	Model	*d*	SE	*Z*	*Q*	*p*
ASD vs. typically developing youth							
Parent + child	64	Fixed	0.78	.0001	6158.87		<.001
		Random	0.97	.0742	13.13		<.001
		Homogeneity test				5,664,859.99	<.001
Parent	53	Fixed	0.78	.0001	5998.65		<.001
		Random	1.21	.1051	11.54		<.001
		Homogeneity test				6,394,385.12	<.001
Child	33	Fixed	0.91	.0005	1850.99		<.001
		Random	0.65	.0669	9.72		<.001
		Homogeneity test				297,566.26	<.001
ASD vs. clinically referred youth							
Parent + child	35	Fixed	0.23	.0004	590.17		<.001
		Random	0.12	.0696	1.76		.077
		Homogeneity test				942,390.70	<.001
Parent	29	Fixed	0.26	.0004	645.74		<.001
		Random	0.13	.0807	1.62		.105
		Homogeneity test				982,528.51	<.001
Child	12	Fixed	0.11	.0009	127.77		<.001
		Random	0.22	.0675	3.25		.001
		Homogeneity test				49,966.92	<.001

It was found that youth with ASD had higher anxiety levels compared to typically developing children. This finding was consistent across models (fixed and random) and respondents (parent and child report), see Table 2. Mean age was not found to moderate the difference in anxiety levels between children with ASD and typically developing youth (*β* = 0.11, *Z* = 0.95, *p* = .340), however, mean IQ was found to be significant (*β = *0.33, *Z* = 4.48, *p* < .001). This result indicates that the difference in anxiety levels between youth with ASD and typically developing youth is related to IQ; that is, as the mean level of IQ of youth with ASD increases, the difference between ASD and typically developing youth in anxiety levels increases as well.

Youth with ASD tend to have higher anxiety levels compared to clinically referred youth, but the results are not consistent, see Table 2. For the fixed effect model a (small) significant difference between ASD and clinically referred youth was found, while for the random effect model this difference was not significant. In addition, child report yielded a significant difference for both the fixed and random effect model, while for parent report the fixed effect model only (and not the random model) indicated a significant difference. Mean age was found to be a significant moderator (*β = *0.33, *Z* = 2.34, *p* = .019), however mean IQ was not (*β = *0.30, *Z* = 1.35, *p* = .178). It was found that as age increased, so did the difference in anxiety levels between ASD and clinically referred youth.

Table 3 displays the results of the meta-analyses comparing the anxiety levels of children with ASD to various clinical comparison groups. It was found that children with ASD have higher anxiety levels compared to youth with externalizing or developmental problems, but not compared to youth with internalizing problems. Furthermore, the results for the comparison between ASD and youth with internalizing problems are inconsistent across models. That is, no significant difference between youth with ASD and youth with internalizing problems was found for the random effect model, while youth with internalizing problems had significantly higher anxiety levels (small effect) than youth with ASD when using the fixed effect model.

**Table 3:** Results of the meta-analyses comparing the anxiety levels of youth with ASD to various clinical groups (based on aggregated parent + child report)

ASD vs.	*k*	Model	*d*	SE	*Z*	*Q*	*p*
Internalizing	12	Fixed	−0.21	.0009	−246.90		<.001
		Random	−0.45	.3302	−1.36		.175
		Homogeneity test				1,310,505.96	<.001
Externalizing	9	Fixed	0.20	.0015	136.78		<.001
		Random	0.38	.1311	2.89		.003
		Homogeneity test				54,716.59	<.001
Developmental	14	Fixed	0.43	.0006	769.95		<.001
		Random	0.32	.0690	4.65		<.001
		Homogeneity test				109,196.76	<.001

## Discussion

This study compared the anxiety levels of youth with ASD to typically developing children and clinically referred children using a meta-analytical approach. Most important findings are that: (1) anxiety levels of youth with ASD are much higher compared to typically developing children (large effect size difference); (2) anxiety levels of children with ASD seem elevated compared to clinically referred children in general (small effect size difference); (3) the type of comparison group seems to matter in the direction that anxiety levels in youth with ASD were found to be higher compared to youth with externalizing or developmental problems, but compared to children with internalizing problems results were inconsistent for the fixed and random model; (4) as IQ increases, so does the difference in anxiety levels between ASD and typically developing children, and (5) as age increases, so does the difference in anxiety levels between ASD and clinically referred children.

The finding that youth with ASD have higher anxiety levels compared to typically developing children is hardly surprising. However, youth with ASD also tend to have higher anxiety levels compared to clinically referred youth (with a small to medium effect size). Therefore it seems that children with ASD are more prone for anxiety problems than other—typically developing and clinically referred—children. Different explanations for these findings have been suggested by various authors (see Bellini 2006, who propose a model for the development of social anxiety in ASD; Wood and Gadow 2010, who propose a model in which ASD and anxiety exacerbate each other; van Steensel et al. (2014) who do not propose an explicit model for the development of anxiety in ASD but do consider other family factors (e.g., parental stress/anxiety, parenting behaviors), next to genetic and ASD-related factors). Taken together, it is thought that children with ASD have a neurobiological predisposition which cause ASD-related difficulties which, in combination with environmental factors (bullying, parenting, etc.), may lead to anxiety. However, more (longitudinal) research, examining both ASD-specific (e.g., theory of mind deficits, severity of ASD, executive functioning problems, social skills deficits) as well as more general factors (e.g., stressful life-events, parental anxiety, parental rearing behaviors) is needed to understand this complex co-occurrence of anxiety and ASD.

It was found in this meta-analysis that the difference in anxiety levels between children with ASD and typically developing children increased when children with ASD had higher IQs, and that the difference in anxiety levels between children with ASD and clinically referred children increased with age. These results are consistent with other studies demonstrating that anxiety in ASD is related to an older age and higher levels of cognitive functioning (e.g., Eussen et al. 2013; Gadow et al. 2005; Mayes et al. 2011). It may be that older children with ASD, and children with ASD who have higher levels of cognitive functioning, are more aware of their difficulties. In combination with possibly higher demands of their surroundings to adapt or to ‘fit in’, this may lead to more stress and higher levels of anxiety. Therefore, especially high-functioning adolescents with ASD may be at risk for developing anxiety disorders and it may be worth to carefully follow and monitor children with ASD transcending to adolescence.

### Limitations

First, significant heterogeneity across studies was found for all meta-analyses. An attempt was made to explain this heterogeneity by including moderators (IQ and age) and by subdividing the clinical comparison groups. Although these variables explained some of the heterogeneity, it still remained significant in all models. Other methodological factors may account for this heterogeneity; e.g., the use of different ASD-samples (e.g., community-based vs. clinically referred), or the different instruments that were used across studies (see Table 1) to assess anxiety (e.g., anxiety specific questionnaire vs. general behavioral questionnaire). In addition, the type of informant (parent vs. child-report) may have played a role. That is, the use of parent-report seems to lead to larger differences in anxiety levels between children with ASD and their comparison group than the use of child reports (see Table 1).

Second, most studies used questionnaires that were not specific for ASD (Table 1). Although this was needed to make a valid comparison to the typically developing children or to the clinically referred youth, it may also have a risk for potential measurement bias. That is, symptoms of ASD and anxiety are not always easy to disentangle and when parents need to fill in a questionnaire there is no way of knowing whether a particular symptom (e.g., ‘is afraid to be among unfamiliar people’) needs to be seen as related to ASD or to anxiety. Due to this mix-up of symptoms, children with ASD may automatically score higher on those questionnaires and cutoffs of traditional anxiety questionnaires may need to be adjusted (e.g., van Steensel et al. 2013). In addition, it is questionable whether the construct of anxiety is similar in youth with ASD. That is, the study of White et al. (2015a) examined the structure of the Multidimensional Anxiety Scale for Children (MASC) with and without ASD and found that while the same factors were found, the levels and relations among factors were found to be different. On the other hand, some traditional measures seem to work quite well for capturing anxiety in (high-functioning) youth with ASD in terms of clinical relevance, psychometric properties (validity, reliability) and sensitivity to change (see Lecavalier et al. 2014).

Third, this meta-analysis demonstrated that the type of comparison group is a relevant factor to consider, however, the number of studies comparing children with ASD to a specific clinical comparison group (e.g., youth with externalizing problems, youth with internalizing problems, etc.) was rather small and too small to conduct analyses separately for child and parent report. In addition, results regarding the comparison between children with ASD and children with internalizing problems were inconsistent across the fixed and random models. That is, while the fixed effects model yielded a (small) significant difference in the direction that youth with internalizing problems had higher anxiety levels, this difference was not found in the random model (indicating similar anxiety levels). Therefore, more research comparing groups of children with ASD to specific clinical samples is needed to draw more definite conclusions.

Finally, a moderating influence of age and IQ was found in this meta-analysis, however, most studies used ASD groups with normal levels of cognitive functioning and with mean ages ranging between 8 and 14 (Table 1). Therefore, future research should focus on both very young children with ASD (<6 years) as well as adolescents (>15 years), and children with lower functioning IQs (<70), as they seem to be somewhat under-represented to date.

### Implications

Given the findings of this study—which demonstrate elevated anxiety levels in youth with ASD—it seems important to develop appropriate screening instruments for anxiety in ASD, in particular as treatment options (e.g., cognitive behavioral therapy) are becoming available and are found to be effective to decrease anxiety in youth with ASD (see meta-analysis of Sukhodolsky et al. 2013). Nowadays, instruments to measure anxiety in ASD are not specifically developed for children with ASD and although the psychometric properties of these instruments seem acceptable for youth with ASD (see review of Lecavalier et al. 2014), for most of these instruments appropriate cutoffs for ASD are lacking. In addition, it is a challenge to distinguish anxiety from ASD. Noteworthy is the study of Ozsivadjian et al. (2012) which examined anxiety triggers and the impact of anxiety symptoms in children with ASD in a focus group. They found evidence for both general anxiety triggers and symptoms (e.g., social worries, specific phobias, avoidance) as well as some ASD-specific triggers and symptoms (e.g., the intensity and pervasiveness of anxiety, sensory sensitivities, social difficulties). Furthermore, the study highlights the impact of anxiety for the daily functioning—not only for the children themselves, but also for the family. It was even noted that the anxiety might have a greater impact on the family life than the ASD, resulting in a further decrease in quality of life (Ozsivadjian et al. 2012). In accordance, the study of van Steensel et al. (2012) demonstrated that anxiety problems in youth with ASD have a negative impact on quality of life, over and above the ASD symptoms. Taken together, the high levels of anxiety in youth with ASD found in this meta-analysis—and the impact of anxiety on the daily functioning of individuals with ASD as demonstrated by previous studies—stresses the importance to gain more insight in the nature and development of anxiety in youth with ASD, as well as the importance of developing appropriate measures and (prevention) treatments.
